# Estimation to Intuition: How Digital Tools Support Diet & Weight Control in Middle & Older Adults

**DOI:** 10.1093/geroni/igaf122.2384

**Published:** 2025-12-31

**Authors:** Sarah Hubner, Julie Blaskewicz Boron

**Affiliations:** Oregon Health Authority, Portland, Oregon, United States; University of Nebraska Omaha, Omaha, Nebraska, United States

## Abstract

Weight dysregulation is associated with increased morbidity and mortality across the lifespan. Age-related changes make weight control more challenging for middle-aged/older adults, highlighting the importance of self-management. Improving dietary balance may help some adults regulate weight; however, making healthy food choices remains difficult. Various methods/tools aim to facilitate healthier eating, including calorie information on menus (CI) and real-time calorie summation (CS) tools. Although understudied in aging adults, CS tools may be especially useful in digital environments for reducing healthy eating barriers (e.g., cognitive load, health literacy). This study explored CI/CS use, expanding on Gustafson & Zeballos (2019). Participants (N = 529; MAge = 56.1 ± 8.4, Range = 45-80; Female = 66.9%) completed an online survey, building and ordering a hypothetical sandwich, then blindly estimating its total calories. Participant condition was randomized (CI, n = 264; CS, n = 265). All participants were provided with calories-per-sandwich-ingredient; however, CS-participants could also view an auto-updating calorie total. Results revealed actual and estimated calories were positively correlated (r = 0.54, p < 0.001) and significantly different (t = 9.9, p < 0.001). Comparing conditions, CI-participants ordered 15.4 more calories (CI = 606.2, CS = 590.8; t = 1.2, p = 0.24), but estimated 35.1 fewer (CI = 510.9, CS = 546.0; t=-2.2, p < 0.001). This led to nearly double the absolute calorie discrepancy (CI = 164.9, CS = 86.3; t = 7.6, p < 0.001). CI-participants also underestimated calories more frequently (CI = 71.6%, CS = 47.9%). Despite not significantly reducing calories ordered, the CS tool improved estimation accuracy, suggesting it may increase awareness in food choice scenarios. While CS tools may not prompt immediate changes to habit/diet, improving calorie estimation could foster intuitive eating. Future research should explore ways to engender this critical skill in middle-aged/older adults, as it may help individuals regulate weight, mitigate disease, and improve aging outcomes.

